# Brexpiprazole Attenuates Aggression, Suicidality and Substance Use in Borderline Personality Disorder: A Case Series

**DOI:** 10.3390/medicina60020283

**Published:** 2024-02-07

**Authors:** Benedict Francis, Vijay A/L Ganasan, Abdul Rasyid Bin Sulaiman

**Affiliations:** 1 Department of Psychiatry, Faculty of Medicine, University of Malaya, Kuala Lumpur 50603, Malaysia; 2 Department of Psychiatry and Mental Health, Hospital Tuanku Ja’afar, Seremban 70300, Malaysia; vijay.ganasan@gmail.com; 3 Division of Medicine, International Medical University, Kuala Lumpur 57000, Malaysia; abdul_rasyid@imu.edu.my

**Keywords:** brexpiprazole, borderline personality disorder, suicide, aggression, substance-related disorders

## Abstract

*Background*: Borderline personality disorder (BPD) is a heterogeneous and highly comorbid disorder. Suicidality, aggression and substance abuse are common presentations of BPD. Our case series is the first to highlight the role of brexpiprazole in improving these symptoms in patients with BPD. *Case presentation*: We describe three cases demonstrating the role of brexpiprazole in improving BPD’s prominent features and comorbidities. All cases improved when brexpiprazole was added to their treatment regime. Case 1: A 26-year-old woman who was diagnosed with BPD and cyclothymia, presented to the psychiatric emergency unit with impulsive suicidal behaviour. Case 2: A 43-year-old woman suffering from BPD sought help due to her violent behaviour and emotional dysregulation. Case 3: A 22-year-old woman with underlying attention deficit and hyperactivity disorder, polysubstance use disorder and BPD presented with dysregulated emotions. *Conclusions*: Our case series provides anecdotal evidence of the potential role of brexpiprazole in attenuating suicidality, aggression and substance abuse in patients with BPD. We postulate that brexpiprazole’s high affinity for the 5HT1A/5HT2A receptors, coupled with its low intrinsic effect on the D2/D3 receptor system, is fundamental in its actions to stabilise the aberrant dopaminergic and serotonergic signalling in BPD. Future research should focus on well-designed clinical trials investigating the efficacy of brexpiprazole in patients with BPD.

## 1. Introduction

Borderline personality disorder (BPD) is a debilitating and highly stigmatised disorder that is characterised by emotional dysregulation, an unstable sense of self, recurrent self-harming attempts, impulsivity and an intense sense of abandonment [1]. Given the dramatic and highly unpredictable presentation of an individual, it is unsurprising that BPD is a major contributor to hospitalisation and psychiatric morbidity in general. The often cited prevalence data of BPD being between 0.7% and 2.7% in the general population may be underestimated [2], as data are scarce from developing countries, particularly the Asian and African regions [3]. Observational data indicate that less than a quarter of patients hospitalised for a self-harm episode suffer from BPD [4]. In addition, around 3% of female inmates at a substance rehabilitation centre were diagnosed with BPD [5].

BPD is also known to be highly comorbid with several primary diagnoses, such as depression [6], bipolar disorder [7], substance use disorder [8], post-traumatic stress disorder [9], and anxiety disorders [10]. The conflation of symptoms between BPD and these Axis 1 conditions further adds to the diagnostic confusion. One might argue that comorbidity is a rule rather than an exception in BPD, as almost 90% of them suffer from another psychiatric condition [11]. For example, the rates of co-occurrence of BPD and bipolar disorders range between 20 and 50%, with higher prevalence noted in community samples [12]. BPD and bipolar disorders share similar symptom domains, such as impulsivity, emotional dysregulation, elated mood, suicidal tendencies and substance abuse, leading to the idea that both disorders are multidimensional, evolving and may lie on a spectrum [7,13,14]. This spectral understanding of BPD has led to interesting pharmacological interventions that run the gamut from mood stabilisers to atypical antipsychotics, including novel partial agonists [15,16]. As BPD is highly heterogeneous, it might be helpful to deconstruct this condition into significant presenting symptoms. In the authors’ collective clinical experience, suicidality, aggression and substance abuse are ubiquitous manifestations of BPD and, thus, critical therapeutic targets. Poor impulse control is the common substrate binding this symptom cluster, resulting from a need for instant over delayed gratification [17]. This cognitive inflexibility may reflect an underlying aberrant incentive salience mechanism in patients with BPD.

Partial agonists across dopaminergic and opioidergic systems have been used with some success in alleviating the symptoms of BPD. For example, there is anecdotal evidence for the use of buprenorphine [18] and some randomised clinical trials examining the efficacy of aripiprazole in the treatment of BPD [19,20,21]. Their success seems to hinge on the ability to modulate and stabilise neurotransmitters implicated in the symptom domain of BPD. Brexipirazole is a novel partial agonist approved by the United States Food and Drug Agency (FDA) in 2015 for the treatment of schizophrenia and adjunctive use in depression [22]. It is a partial agonist at the D2, D3 and 5HT1A receptors and a full antagonist at the 5HT2A, 5HT2B, 5HT7 and noradrenergic α receptors [23]. Relative to cariprazine and aripiprazole, brexpiprazole has the highest affinity to the 5HT1A receptor (Ki: 0.12 nM) [24], thus enabling it to modulate emotional dysregulation and impulsivity seen in BPD patients.

Brexpiprazole has low D2 intrinsic activity with the highest D2 binding affinity relative to other partial agonists, translating to an optimal and stabilised dopaminergic tone (brexpiprazole Ki: 0.30 nM; aripiprazole Ki: 0.34 nM; cariprazine Ki: 0.49 nM) [25]. In the presence of high endogenous dopamine, brexpiprazole acts as a net dopamine antagonist; conversely, it acts as an agonist when dopamine levels are low. This ability to modulate the dopaminergic transmission can avoid the side effects of excessive D2 receptor activation, such as extrapyramidal side effects and hyperprolactinemia, while maintaining its antipsychotic properties. A corollary to this idea is that the partial agonism of brexpiprazole avoids the phenomenon of dopamine receptor upregulation and, thus, supersensitivity psychosis, even with repeated administration [26]. A small, proof-of-concept study previously described the efficacy of brexpiprazole in reducing symptom severity in BPD [27]. However, high placebo response rates and the survey’s short duration failed to show the separation of efficacy between brexpiprazole and placebo response. In addition, the authors measured BPD symptom severity in totality and did not highlight specific symptom domains affected by brexpiprazole. To the authors’ knowledge, this is the first case series to describe the role of brexpiprazole in attenuating the domains of suicidality, aggression and substance abuse in BPD.

## 2. Case Description

### 2.1. Case 1

A 26-year-old woman presented to the psychiatric emergency unit with intense low mood and suicidal ideations. On the day of admission, she told her friend that she wanted to end her life by jumping off a balcony. She had seen a psychiatrist in the past for depression but was never compliant with treatment. She suffered from low mood for several years but had worsening depressive symptoms the past year due to her ailing mother, who was suffering from terminal cancer. Subsequently, she had heated debates with her brother about who would be the mother’s caregiver, given her frail state. These arguments caused her much mental anguish, and she descended into a whirlwind episode of depressed mood, temper tantrums, poor concentration, increased appetite, loss of interest in her surroundings and dissociative episodes. She described the dissociative episodes as transient but surreal experiences of depersonalisation and ‘being a visitor in my own body’. She also admitted to engaging in deliberate self-harming to relieve her emotional numbness. For example, she would ingest soap and cut her wrist repeatedly in a superficial manner when she was upset with herself. Recently, her self-harming tendencies became more regular, resulting in multiple healing scars on both her forearms. She would also resort to binge drinking alcohol until she became intoxicated as a coping strategy. She was admitted to the inpatient unit for observation.

Further history revealed a background of a traumatic and abusive childhood, constant bullying in school and a deeply ingrained sense of low self-esteem. Over the years, there were times when she exhibited sub-syndromal hypomanic symptoms, characterised by impulsive online gambling, anger outbursts towards family members, reduced need for sleep and being sexually disinhibited. Psychodynamic psychotherapy uncovered deep scars of unresolved trauma and internalised anger towards her parents for dismissing the sexual assault by her uncle when she was an adolescent.

After formulating her case, a diagnosis of BPD with comorbid cyclothymia was made. She was initiated on regular psychodynamic psychotherapy in the ward, alongside oral quetiapine 50 mg BD and escitalopram 5 mg OD. After a week and a half, she reported emotional numbness, passive death wishes and excessive daytime sedation. Additionally, the nurses observed that she was irritable and kept herself isolated. The treating clinician decided to stop the escitalopram and cross-taper the quetiapine with brexpiprazole, which was started at 1 mg OD and titrated up to 2 mg after a week. A week later, she reported feeling less suicidal and had no emotional lability. Her mood was less depressed, and she had good mental clarity. In the third week, she was no longer suicidal and had mood reactivity to positive stimuli. She was discharged from the ward and given an outpatient follow-up appointment. During her subsequent clinic visit, she was in remission of her symptoms and had not engaged in self-harming episodes. Her brother eventually reconciled with the patient, enhancing her response to treatment. She is now in functional remission with brexpiprazole 2 mg OD without any suicidal ideations and did not report any adverse effects (Table 1).

### 2.2. Case 2

A 43-year-old female arranged a psychiatric consult to discuss her emotional lability in her marriage. She was in a tumultuous relationship with her husband, having been married for the past 20 years. She described her relationship with her husband as strained as she would often lose her temper with him and, at times, attack him with a baton they kept at home. As a result, they were involved in several physical altercations, leading to her husband being kicked and punched in the face. She was embarrassed at her sudden outbursts of anger but blamed her dissociative self whenever she was in a rage. Her husband added that she would also scratch and slap herself when she was angry, eventually resorting to binge eating. During these episodes, she would overeat for several days and drink high-caloric fluids. Arguments in her marriage usually precipitated these episodes. In addition, she had periods of emotional dysregulation characterised by irritability, euphoria and depressive symptoms. Several years earlier, she saw a psychiatrist who formulated that the binge eating episodes, temper tantrums and low mood were part of her poor impulse control. She was started on fluoxetine 20 mg OD, which was optimised to 60 mg OD after two months. About six months ago, she was also started on oral quetiapine 100 mg BD to help with her aggressive tendencies, but to no avail. She did not have significant hypomanic or manic symptoms.

Regarding her previous history, she had similar patterns of violence in previous relationships. She would impulsively attack her partner whenever they had a misunderstanding. During periods of stress, she would accuse the husband of infidelity and sleeping with prostitutes. It was evident that she projected her low self-esteem in the form of paranoia and violence towards her husband. She was also deeply insecure and would become defensive whenever her husband pointed out her mistakes in the marriage. Her husband also reported that she had frequent dissociative episodes whereby she would appear to be in a trance and cut her arms with a butter knife. A psychodynamic formulation of her history revealed that her father physically abused her as a child, and she often witnessed him slapping her mother after an argument. As a young adult, she sank into a deep depressive episode whereby she experienced intense depressive symptoms coupled with a solid intent to commit suicide. Her stressor at the time was a failed relationship after her partner cheated on her. She was admitted to the ward and discharged with escitalopram 10 mg OD. However, she was not compliant with the treatment. A few years later, in her late twenties, she developed binge eating and purging behaviour to cope with her low self-esteem and inner frustrations toward men. She underwent structured psychotherapy for a short while, which helped reduce her binges.

After analysing her history, the psychiatrist revised her diagnosis to BPD. At this point, she was already on fluoxetine 60 mg OD and quetiapine 100 mg BD; however, she showed a slight improvement in her mood and anger management. After two weeks, her quetiapine was then increased gradually to 300 mg BD, and her fluoxetine was tapered off due to lack of efficacy. However, she complained of constipation and sedation with the increased dose of quetiapine. The psychiatrist then reduced her quetiapine back to 200 mg BD and introduced brexpiprazole 1 mg OD to help with her aggressive tendencies and impulsivity. After two weeks, she felt less agitated, and her husband reported fewer explosive anger episodes at home. The brexpiprazole was increased to 2 mg OD and eventually to 3 mg OD after two weeks. She developed transient nausea and headaches with brexpiprazole 3 mg OD, which resolved at week 3 of treatment. She felt more in control of her anger and could follow through with her mindfulness exercises to remain grounded when upset. Her mood also perked, and she had fewer episodes of self-harming behaviour. She regained her interest in gardening and rejoined her cooking classes with her friends. She also agreed to join marital counselling with her husband to rejuvenate their relationship. After two months, she reported a stable mood and did not have any new aggressive tendencies at home. Her binge eating episodes reduced significantly, with improvement in her BPD symptomatology. Her mood, eating habits and BPD symptomatology are now stable on quetiapine 200 mg BD and brexpiprazole 3 mg OD (Table 1).

### 2.3. Case 3

A 22-year-old female with underlying Attention Deficit and Hyperactivity Disorder (ADHD) and substance-use disorder presented with anger and frustration towards her boyfriend after she discovered he was in an adulterous relationship. They had been dating for one year, and his infidelity caused her great emotional distress. She reacted by smoking cannabis heavily, engaging in self-harming behaviour by cutting her thighs, and binge drinking alcohol. She was also emotionally dysregulated at home, screaming impulsively at her family members when she was upset. Further exploration of her history revealed that she exhibited attention-deficit symptoms since the age of 14 years old. Teachers observed that she was fidgety and playful in school and liked to procrastinate in her work. Her abuse of substances started in her early teenage years as a form of self-treatment for her ADHD symptoms. She abused cannabis, heroin and methamphetamine both at school and at home. She was caught several times smoking cannabis in class and threatened with expulsion. Her adolescent years were characterised by a chaotic upbringing, coloured by the constant bickering between her parents. Her father was largely absent, while her mother was too emotionally distressed to handle her adolescent mood swings. At the age of 17, her parents divorced, and her father remarried soon after, further compounding her emotional turbulence. Her cannabis and methamphetamine abuse also increased during this time. She also had multiple short-lived relationships with both men and women, which she described as a coping mechanism. However, these relationships were transient, as she was possessive, insecure and volatile. She admitted to fearing abandonment, resulting in her being overdependent on her partners. In addition, she began self-harming by slapping herself and drinking small amounts of soap fluid whenever she was distressed. These actions brought her some emotional reprieve. She consulted a psychiatrist at this point and started on strattera 40 mg OD, desvenlafaxine 50 mg OD and naltrexone 50 mg OD. These medications helped with her focus and attention; however, they did not quell her craving for cannabis and methamphetamines. Her naltrexone was tapered up gradually to 150 mg OD after a month with little impact on her cravings.

Upon reviewing her at our clinic, the treating team reformulated her diagnosis to BPD with comorbid ADHD and polysubstance use disorder. The psychiatrist augmented her treatment regime with aripiprazole 15 mg OD for three weeks and later switched to lurasidone 40 mg OD due to the lack of efficacy of the latter. However, both atypical antipsychotics failed to induce remission of her symptoms. Finally, the treating psychiatrist decided to switch her antipsychotic to brexpiprazole, which was initiated at 1 mg OD and tapered up to 3 mg OD. After three weeks, she started gaining more control over herself and was willing to work on coping mechanisms. By the end of four weeks, her self-harming behaviour had reduced, and her emotions were regulated. She did not experience explosive bouts of anger and could remain calm when triggered. Notably, she did not experience any akathisia with the addition of brexpiprazole. She stayed sober and said that her cravings were manageable. She was also open to initiating Motivational Interviewing for her substance use issues. Her mother said she was less impulsive and did not have explosive bouts of anger with brexpiprazole 3 mg OD, straterra 40 mg OD and naltrexone 150 mg OD (Table 1).

**Table 1:** Summary of patient characteristics.

Patient	Diagnosis	Presentation	Medication	Adverse Effects
1	BPD and Cyclothymia	Suicidality and emotional dysregulation	Brexpiprazole 2 mg OD	None
2	BPD	Aggressive tendencies, binge eating episodes, and low mood	Brexpiprazole 3 mg OD + quetiapine 200 mg BD	Transient nausea and headache
3	BPD, ADHD and substance use disorder	Polysubstance abuse, ADHD symptoms and self-harming behaviour	Brexpiprazole 3 mg OD + straterra 40 mg OD + naltrexone 150 mg OD	None

## 3. Discussion

BPD remains a challenging diagnosis due to the heterogeneity of its presentation. Indeed, the presentation of a person living with BPD is exceptionally varied, including recurrent suicidality, deliberate self-harm, eating disorder issues, aggression, dissociation, substance abuse, mood dysregulation and intense interpersonal rejection [28]. Several key symptoms of BPD are linked to amygdala hypersensitivity and prefrontal hyporeactivity mediated by dysfunctional serotonergic and dopaminergic transmission [29]. In this case series, the authors highlight the role of brexpiprazole in ameliorating three symptom domains of BPD, namely suicidality, substance abuse and aggression. The common neurobiological denominator of these presentations is the uneven stimulation of the 5HT1A receptor, affecting the overall serotonergic tone at the prefrontal and limbic areas of the brain [30]. Partial agonists, notably brexpiprazole, may mediate effective symptomatic relief in BPD due to the high binding affinity for the 5HT1A receptor.

One of the patients depicted in the case series exhibited intense aggressive tendencies. The serotonin deficiency hypothesis states that aggression is a function of reduced serotonergic levels in the brain. Preclinical studies have shown an overactivity of 5HT1A autoreceptors and under-stimulation of the postsynaptic 5HT1A receptors in aggressive behaviour [31]. Thus, modulating serotonergic tone is a possible therapeutic option for managing aggression in BPD patients. Brexpiprazole’s high 5HT1A/5HT2A affinity ratio translates to a net effect of serotonergic tone in the cortico-limbic areas while avoiding the activating effects of increased dopamine as a result of its low intrinsic activity at the D2 and D3 receptors. Among the antipsychotic partial agonists, brexpiprazole is the most serotonergic, having the highest binding affinity for the 5HT1A receptor (k*_i_*: 0.12 nM) [32]. The authors postulate that this 5HT1A-preferring action of brexpiprazole is crucial for its efficacy in alleviating aggressive tendencies in BPD.

The first patient depicted in our case series had intense suicidal ideations as part of her BPD symptomatology. Indeed, suicidality is a common and recurrent theme in BPD and is hypothesised to be due to the paucity of postsynaptic 5HT1A in the raphe nucleus and an adaptive reduction in 5HT1A receptors autoreceptors to increase serotonergic projections in patients who committed suicide [33]. Reduced 5-hydroxyindolacetic acid (5-HIAA) and Homovanellic Acid (HVA) in the cerebrospinal fluid of suicidal patients indicate that there are lowered levels of these monoamines in the brains of suicidal patients [34]. The authors postulate that brexpiprazole’s ability to act as a functional agonist of 5-HT1A and D2/D3 receptors in low endogenous monoamine states renders it capable of strengthening striatal dopamine and serotonin projections, thus improving suicidality.

Substance use in BPD patients is a frequent cause of increased disease morbidity. Evidence shows that almost 60% of patients with BPD also have a comorbid substance use disorder, leading to increased psychopathology and, ultimately, suicidal behaviour [35]. In the context of comorbid ADHD, as with the third patient, patients with BPD are more inclined to abuse substances. The rates of comorbid ADHD in patients with BPD have been reported to be as high as 60% [36]. It is interesting to note that the genome-wide association studies (GWAS) data showed no particular overlap for BPD and ADHD [37]. However, a novel Swedish study found that individuals within the same family who had ADHD were 19.4 times more likely to develop concomitant BPD [38]. Thus, shared epigenetics and environmental factors might play a role in the development of this association. Emotional dysregulation and poor impulse control are the core symptoms of both ADHD and BPD. Substance intake, particularly stimulants, may modulate the dopaminergic dysfunction seen in these disorders [39] and thus be abused as a form of self-medication.

Partial agonists such as aripiprazole and cariprazine have both been used to good effect in patients with dual diagnosis [40,41,42,43]. Brexpiprazole was effective in our third case as it reduced the patient’s craving and problematic polysubstance intake. The authors’ experience aligns with preclinical evidence showing the efficacy of brexpiprazole in reducing opioid-seeking behaviour in mice by stabilising the limbic dopaminergic tone [44]. Substance use and withdrawal are a function of dopaminergic flux; repeated usage leads to dopaminergic surges, while withdrawal is linked to a dopaminergic trough, resulting in cravings. Through their action on the D2/D3 and 5HT1A receptors, partial agonists ensure optimal dopamine levels in the striatum to soothe cravings while preventing reinforcement of drug-seeking behaviour. Brexpiprazole might have an edge over other atypical antipsychotics in treating BPD patients with dual diagnosis because of its relatively low propensity to cause akathisia [45]. Indeed, psychoactive substances may cause increased presentation of extrapyramidal symptoms such as akathisia, parkinsonism, and tardive dyskinesia. The authors postulate that treatment with a partial agonist antipsychotic with a low propensity for akathisia may thus be beneficial for this group of patients.

The usage off-label usage of brexpiprazole needs to be prefaced with a word of caution, particularly in terms of potential side effects. Common side effects of brexpiprazole include akathisia, headache, fatigue, sleepiness, constipation and tremors. These side effects might be compounded in patients with BPD as they are often on polypharmacy, potentially leading to problematic drug–drug interactions. However, it should be noted that brexpiprazole’s balanced D2/5HT1A binding profile translates to low extrapyramidal and other D2-mediated side effects [46].

## 4. Conclusions

An essential preliminary recommendation that can be derived from our case series is that brexpiprazole has the potential to alleviate suicidality, aggression and substance use in BPD due to its 5HT1A-preferring nature, coupled with low intrinsic D2/D3 action. The study’s primary limitation is that it is anecdotal and describes the authors’ clinical experience in their real-world practice. Thus, the impact of brexpiprazole on BPD cannot be inferred from this study. Future research should investigate the efficacy, safety and tolerability of brexpiprazole as a treatment for BPD patients in an adequately powered randomised controlled trial design.

## Data Availability

Data are contained within the article.
